# Association between heavy metals, essential trace elements in follicular fluid and diminished ovarian reserve: a hospital-based case-control study

**DOI:** 10.3389/fendo.2026.1889096

**Published:** 2026-07-02

**Authors:** Xin-chen Huang, Ming-li Sun, Yi-qin Chen, Xin-lan Zhang, Xin-yao Song, Wen-xiang Wang, Yan Sun

**Affiliations:** 1 School of Basic Medical Sciences, Fujian Medical University, Fuzhou, Fujian, China; 2 College of Clinical Medicine for Obstetrics & Gynecology and Pediatrics, Fujian Medical University, Fuzhou, Fujian, China; 3 Medical Research Center, Fujian Maternity and Child Health Hospital, College of Clinical Medicine for Obstetrics & Gynecology and Pediatrics, Fujian Medical University, Fuzhou, China; 4 Department of Health Inspection and Quarantine, School of Public Health, Fujian Medical University, Fuzhou, Fujian, China; 5 Center of Reproductive Medicine, Fujian Maternity and Child Health Hospital, College of Clinical Medicine for Obstetrics & Gynecology and Pediatrics, Fujian Medical University, Fuzhou, China

**Keywords:** diminished ovarian reserve, essential trace elements, follicular fluid, heavy metals, mixture effects

## Abstract

**Objective:**

To investigate the distribution of heavy metals and essential trace elements in follicular fluid (FF) and their association with diminished ovarian reserve (DOR) using a clinical epidemiological approach.

**Methods:**

A hospital-based case–control study was conducted. Following propensity score matching (PSM) to control for demographic confounders (age and BMI), baseline clinical data and FF samples were analyzed. Inductively coupled plasma mass spectrometry was used to quantify metal elements in FF. Associations were evaluated using Spearman correlation analysis, weighted quantile sum (WQS) regression, Bayesian kernel machine regression (BKMR), and least absolute shrinkage and selection operator (LASSO) regression.

**Results:**

FF concentrations of arsenic (As), strontium (Sr), and calcium (Ca) were significantly higher in the DOR group than in the control group (*P*_(As)_ = 0.001, *P*_(Sr)_ < 0.001, *P*_(Ca)_ = 0.002). Spearman correlation analysis revealed significant positive correlations between As and selenium, Sr, and vanadium (V). Logistic regression demonstrated that As (odds ratio [*OR*] = 4.727, 95% confidence interval [*CI*]: 2.456–9.611) and Sr (*OR* = 1.984, 95% *CI*: 1.350–2.985) were associated with higher odds of DOR, whereas copper (*OR* = 0.552, 95% *CI*: 0.359–0.831), V (*OR* = 0.366, 95% *CI*: 0.183–0.716), and zinc (Zn) (*OR* = 0.497, 95% *CI*: 0.308–0.789) were associated with lower odds of DOR. The WQS regression identified Sr, As, and Ca as the primary contributors to the mixture (weights: 0.398, 0.302, and 0.277, respectively). The BKMR model suggested a positive but relatively weak overall mixture effect of metal exposure on the likelihood of DOR. In the advanced mixture model, As, Ca, and iron (Fe) were positively associated with DOR, whereas Zn was negatively associated with DOR, with V and Sr no longer retained as independent factors.

**Conclusion:**

Heavy metals and essential trace elements in FF exhibit joint mixture effects in the pathogenesis of DOR. While Sr serves as a surrogate marker for cumulative exposure, As, Ca, and Fe are independently and positively associated with the likelihood of DOR, whereas Zn exhibits a protective association. Maintaining local trace element homeostasis may be critical for preserving ovarian reserve.

## 1 Introduction

Diminished ovarian reserve (DOR) is a major pathological contributor to the increasing global burden of infertility (1). Among infertile populations, the prevalence of DOR is estimated to be approximately 10%. Notably, both the incidence of DOR and its occurrence at younger ages have shown an increasing trend in recent years (2, 3). The etiology and pathogenesis of DOR remain incompletely understood; however, current evidence suggests that it results from a complex interplay of genetic, metabolic, immunological, infectious, and environmental factors. Clinically, DOR is typically characterized by an insidious onset and progressive decline in ovarian function (4).

Follicular fluid (FF) provides the immediate microenvironment for oocyte growth and development, as well as for the function of ovarian granulosa cells. Exogenous substances, including environmental xenobiotics, can cross the blood–follicle barrier and accumulate within the follicular cavity, thereby potentially impairing follicular development (5, 6). Increasing evidence indicates that metal elements can enter and accumulate in FF (7, 8). These elements are broadly classified into heavy metals and essential trace elements. While earlier studies suggested that certain trace elements may exert beneficial effects on reproductive function (9), more recent evidence highlights their potential adverse effects on ovarian physiology.

Previous studies have investigated the effects of metal elements on ovarian function; however, the findings remain inconsistent. For example, Ingle et al. reported that chromium (Cr) concentrations in FF were negatively correlated with the number of mature oocytes, possibly through the inhibition of oocyte maturation or the induction of apoptosis (10). Granulosa cells play a central role in follicular development; thus, their dysfunction or apoptosis represents a key mechanism underlying ovarian insufficiency (11). Lv et al. demonstrated that cadmium (Cd) exposure disrupts the tricarboxylic acid cycle, calcium (Ca) signaling, and amino acid metabolism in granulosa cells, ultimately inducing apoptosis and impairing ovarian reserve (12). In contrast, Genard-Walton et al. reported no significant association between circulating levels of several heavy metals (lead [Pb], mercury [Hg], Cd, and Cr) and DOR in a cohort study including 139 cases and 153 controls (13).

These inconsistent findings highlight the need for further investigation into the role of metal exposure in ovarian reserve, particularly using well-designed epidemiological studies. Therefore, the present study aimed to evaluate the association between heavy metals and essential trace elements in FF and DOR in women of reproductive age using a case–control design. Both single-element effects and mixture effects were assessed using advanced statistical approaches. The findings of this study may provide new insights into the potential impact of environmental metal exposure on ovarian function and contribute to the development of preventive strategies for infertility.

## 2 Materials and methods

### 2.1 Study design

A hospital-based case–control study was conducted among women with infertility who underwent intracytoplasmic sperm injection (ICSI) at the Assisted Reproductive Technology Research Department of the Fujian Maternity and Child Health Hospital between December 2025 and April 2026. Based on clinical diagnoses and predefined inclusion and exclusion criteria, 195 participants were enrolled in the DOR group. Concurrently, 237 women who met the control criteria and underwent assisted reproductive procedures due to male-factor infertility were recruited as the control group. All participants provided written informed consent prior to enrollment. The study protocol was approved by the Institutional Review Board of Fujian Maternity and Child Health Hospital (Approval No. 2025KY294) and was conducted in accordance with relevant ethical guidelines and regulations.

The selection of women undergoing ICSI for male-factor infertility as the control group was primarily driven by ethical and practical considerations. Obtaining follicular fluid requires controlled ovarian hyperstimulation and invasive transvaginal ultrasound-guided oocyte aspiration. It is ethically unfeasible to perform these invasive procedures on healthy volunteers from the general population solely for research purposes. Therefore, women with a normal ovarian reserve who are undergoing ICSI strictly due to male-factor infertility serve as the most ethically appropriate and clinically accessible proxy for representing a “normal ovarian microenvironment” in reproductive endocrinology research.

### 2.2 Participants

Inclusion criteria:

DOR group: Women aged <40 years who met at least one of the following criteria for DOR: (1) basal follicle-stimulating hormone (FSH) level >10 IU/L measured on menstrual cycle days 2–4; (2) basal FSH to luteinizing hormone (LH) ratio>3; (3) anti-Müllerian hormone (AMH) level <1.0 ng/mL; or (4) basal antral follicle count (AFC) <5.

Control group: Women aged <40 years who met all of the following criteria: (1) normal basal FSH and LH levels; (2) regular menstrual cycles with confirmed ovulation; and (3) absence of infertility attributable to ovarian or endocrine disorders.

Exclusion criteria:

Participants were excluded if they met any of the following conditions: (1) failure to meet the inclusion criteria; (2) history of hyperprolactinemia, congenital adrenal hyperplasia, diabetes mellitus, thyroid dysfunction, Cushing’s syndrome, hypertension, hypercholesterolemia, or malignancy; (3) use of medications within three months prior to enrollment, including insulin-sensitizing agents, oral contraceptives, anti-androgens, statins, aspirin, or corticosteroids; (4) hepatic or renal dysfunction, active malignancy, or use of mineral supplements or diuretics within the preceding three months; (5) major psychiatric disorders; (6) history of prolonged hormone therapy; (7) congenital ovarian dysplasia or history of unilateral or bilateral oophorectomy; or (8) incomplete clinical data or poor compliance with the study protocol.

### 2.3 Clinical data collection

Baseline demographic and clinical characteristics were retrospectively extracted from medical records of patients undergoing ICSI at the Reproductive Center of Fujian Maternity and Child Health Hospital between January 2022 and December 2023. Collected variables included anthropometric parameters (height and weight), age, number of retrieved oocytes, and endocrine profiles. Hormonal measurements included FSH, LH, AMH, prolactin (PRL), estradiol (E2), and testosterone (T).

### 2.4 FF collection

Controlled ovarian hyperstimulation was performed using a conventional long protocol involving a gonadotropin-releasing hormone agonist (GnRH-a), gonadotropins (Gn), and human chorionic gonadotropin (HCG). Pituitary downregulation was initiated during the mid-luteal phase of the preceding cycle or on menstrual cycle day 2. Exogenous Gn were administered starting on menstrual cycle day 3. Follicular development was monitored by transvaginal ultrasonography, and gonadotropin dosage was adjusted accordingly. When at least two follicles reached a mean diameter of ≥18 mm or three follicles reached ≥16 mm, ovulation was triggered by intramuscular HCG (5,000–10,000 IU). Oocyte retrieval was performed 36 hours after HCG administration. Follicles ≥18 mm were aspirated under ultrasound guidance, strictly without the use of any flushing medium to avoid sample dilution. After identification of the cumulus–oocyte complex, the remaining pure FF from multiple dominant follicles of the same patient was pooled and collected under sterile conditions to form exactly one representative sample per participant, thereby ensuring sufficient volume for subsequent multi-element analysis. Blood-contaminated samples, defined by macroscopic visual inspection as having any visible pink or red discoloration prior to centrifugation, were excluded. Samples were centrifuged at 1,000 × g for 5 minutes, and the supernatant was aliquoted and stored at -80 °C until analysis. All samples were collected during the same continuous study period, ensuring that storage durations at -80 °C were comparable between the DOR and control groups. To maintain maximum analytical stability, all sample aliquots strictly underwent a single freeze-thaw cycle at room temperature immediately prior to acid digestion and ICP-MS quantification.

### 2.5 Determination of metal element concentrations in FF

Frozen FF samples were retrieved from -80 °C storage. A 200 μL aliquot was transferred into a microwave digestion vessel, followed by the addition of 3 mL concentrated nitric acid (68% v/v) and 4 mL deionized water. Samples were subjected to microwave digestion using a programmed temperature gradient (100 °C, 130 °C, 160 °C, and 180 °C). After cooling, the digested samples were diluted to a final volume of 10 mL with deionized water. Metal concentrations were quantified using inductively coupled plasma mass spectrometry (ICP-MS). The analyzed elements included heavy metals and metalloids (arsenic [As], Cd, Pb, thallium [Tl], antimony [Sb], beryllium [Be], Cr) and essential trace elements (cobalt [Co], copper [Cu], lithium [Li], molybdenum [Mo], manganese [Mn], nickel [Ni], selenium [Se], strontium [Sr], vanadium [V], zinc [Zn], titanium [Ti], Ca, magnesium [Mg], and iron [Fe]). Instrument parameters were as follows: radiofrequency power, 1300 W; plasma gas flow rate, 15 L/min; nebulizer gas flow rate, 0.97 L/min; and auxiliary gas flow rate, 1.20 L/min. To ensure analytical quality, quantification was performed using multi-point external standard calibration curves demonstrating excellent linearity (R^2^ > 0.999). Although an internal standard was not utilized, potential matrix effects and instrumental drift were effectively minimized through thorough sample digestion and substantial dilution. Furthermore, all samples were injected in a random order to reduce uncertainty caused by variations in injection sequence and instrument sensitivity during the analysis. Measurements were repeated three times for each sample, and the relative standard deviations (RSD) of the results were consistently less than 5%. In the negative control solutions, the target elements were not detected. The limit of detection (LOD) for each element was determined, and the limit of quantitation (LOQ) was defined as three times the LOD. These metrics, alongside the specific spike-recovery percentages for all target elements, are detailed in **Supplementary Table 1**.

### 2.6 Statistical analysis

Statistical analyses were performed using SPSS Statistics (version 21.0) and R (version 3.5.1). Weighted quantile sum (WQS) regression and Bayesian kernel machine regression (BKMR) were conducted using the R packages gWQS (version 1.1.0) and BKMR (version 0.2.0), respectively. Continuous variables were assessed for normality. Normally distributed data were expressed as mean ± standard deviation (SD) and compared using the independent samples t-test. Non-normally distributed data were presented as median and interquartile range (IQR) and analyzed using the Mann–Whitney U test. Spearman correlation analysis was used to assess relationships among metal elements and between metal concentrations and hormonal indicators. Propensity score matching (PSM) was applied to balance age and body mass index (BMI) between groups.

To evaluate the association between metal mixtures and DOR, WQS and BKMR models were used to estimate overall effects, interactions, and individual contributions of each element. Least absolute shrinkage and selection operator (LASSO) regression was used for variable selection. All statistical tests were two-sided, and a *P* value < 0.05 was considered statistically significant.

## 3 Results

### 3.1 Population characteristics

A total of 432 participants were included in this study, comprising 237 individuals in the control group and 195 in the DOR group. Baseline characteristics are presented in **Table 1**. Significant differences were observed between the two groups in terms of age, number of retrieved oocytes, FSH, T, and AMH levels (*P* < 0.05). Specifically, participants in the DOR group were older and had higher FSH levels compared with controls (both *P* < 0.001). In contrast, the number of retrieved oocytes, T levels, and AMH levels were significantly lower in the DOR group (*P*_(oocytes)_ < 0.001; *P*_(AMH)_ < 0.001; *P*_(T)_ = 0.001).

**Table 1:** Baseline characteristics of the participants.

Characteristic	Control (N = 237)	DOR (N = 195)	*P*
BMI (kg/m²)	21.26 (19.44, 22.96)	21.37 (19.82, 23.46)	0.446
Age (years)	31.00 (29.00, 35.00)	35.00 (31.00, 39.00)	<0.001
Number of retrieved oocytes	9.00 (7.00, 12.00)	4.00 (2.00, 6.00)	<0.001
FSH (mIU/mL)	6.08 (5.22, 7.36)	7.51 (6.09, 10.07)	<0.001
LH (mIU/mL)	3.30 (2.60, 4.50)	3.10 (2.30, 4.25)	0.161
PRL (ng/mL)	13.40 (10.15, 19.33)	14.10 (10.15, 19.90)	0.512
E_2_ (pg/mL)	36.00 (28.00, 46.50)	36.00 (25.00, 47.00)	0.792
T (ng/dL)	0.28 (0.22, 0.36)	0.25 (0.20, 0.33)	0.001
AMH (ng/mL)	3.12 (2.18, 4.72)	0.82 (0.53, 1.26)	<0.001

Data are presented as Median (25th Percentile, 75th Percentile).

BMI, Body Mass Index; FSH, Follicle-Stimulating Hormone; LH, Luteinizing Hormone; PRL, Prolactin; E2, Estradiol; T, Testosterone; AMH, Anti-Müllerian Hormone.

### 3.2 Distribution of metal elements in FF

Among the 21 trace elements initially quantified by ICP-MS, 10 elements (Be, Cd, Co, Li, Mo, Mn, Ni, Pb, Sb, and Tl) were excluded from subsequent statistical analyses due to their low detection frequencies and concentrations remaining below the limit of detection (LOD) in the majority of samples. Consequently, our final statistical models focused on the remaining 11 robustly detected elements (**Table 2**). As and Sr concentrations were significantly higher in the DOR group than in the control group (both *P* < 0.001). In contrast, Cu, Zn, Ti, Cr, and Mg concentrations were significantly lower in the DOR group (all *P* < 0.001).

**Table 2:** Exposure levels of heavy metals and essential trace elements in follicular fluid.

Element	Control (N = 237)	DOR (N = 195)	*P*
As (μg/L)	29.59 (25.92, 34.33)	33.01 (29.10, 36.09)	<0.001
Cu (μg/L)	711.09 (592.17, 845.81)	561.98 (420.85, 740.48)	<0.001
Se (μg/L)	71.51 (57.39, 92.81)	72.53 (56.37, 88.04)	0.495
Sr (μg/L)	27.92 (20.99, 35.89)	38.42 (31.22, 45.47)	<0.001
V (μg/L)	27.94 (22.70, 38.19)	29.95 (26.01, 37.19)	0.045
Zn (μg/L)	393.83 (176.43, 478.94)	264.93 (148.76, 381.89)	<0.001
Ti (μg/L)	173.06 (147.00, 200.09)	146.57 (116.98, 182.71)	<0.001
Cr (μg/L)	97.42 (82.80, 118.86)	84.96 (67.06, 104.94)	<0.001
Ca (mg/L)	51.10 (43.90, 71.40)	57.55 (51.84, 65.18)	0.002
Mg (mg/L)	39.88 (33.63, 48.53)	30.23 (23.73, 41.56)	<0.001
Fe (μg/L)	1526.72 (1034.15, 1931.85)	1330.44 (976.16, 1747.99)	0.027

Data are presented as Median (25th Percentile, 75th Percentile).

As, Arsenic; Cu, Copper; Se, Selenium; Sr, Strontium; V, Vanadium; Zn, Zinc; Ti, Titanium; Cr, Chromium; Ca, Calcium; Mg, Magnesium; Fe, Iron.

### 3.3 Correlation analysis of metal elements

Spearman correlation analysis revealed significant inter-element correlations among metal concentrations in FF (**Supplementary Figure 1**). Positive correlations (all P < 0.001) were observed between As and Cu, Se, Sr, V, Zn, and Cr. Cu was also positively correlated with Se, V, Zn, Ti, Cr, Mg, and Fe. Similarly, Se showed positive correlations with Sr, V, Zn, Ti, Cr, Mg, and Fe. Additionally, Sr was positively correlated with V, whereas Zn was positively correlated with Ti, Cr, Ca, Mg, and Fe. Ti and Cr were also positively correlated with Mg and Fe (all *P* < 0.001). In contrast, Sr showed significant negative correlations with Ti, V, and Ca (all *P* < 0.001).

### 3.4 Population characteristics after PSM

Given that age is an important confounding factor for DOR, PSM was performed to balance age and BMI between groups. A total of 121 participants in the DOR group were successfully matched with 121 controls.

Baseline characteristics after matching are presented in **Supplementary Table 2**. FSH levels remained significantly higher in the DOR group (*P* < 0.001), whereas the number of retrieved oocytes, T levels, and AMH levels were significantly lower compared with the control group (*P*_(oocytes)_ < 0.005 for, *P*_(T)_ = 0.005, *P*_(AMH)_ < 0.001).

### 3.5 Metal element levels after PSM

After PSM, As, Sr, and Ca concentrations remained significantly higher in the DOR group compared with controls (*P*_(As)_ = 0.001, *P*_(Sr)_ < 0.001, *P*_(Ca)_ < 0.001). Conversely, Cu, Zn, Cr, and Mg concentrations were significantly lower in the DOR group (all *P* < 0.001). In addition, Ti and Fe levels were also reduced in the DOR group (*P*_(Ti)_ = 0.035, *P*_(Fe)_ = 0.038) (**Supplementary Table 3**).

### 3.6 Correlation analysis after PSM

**Figure 1:**
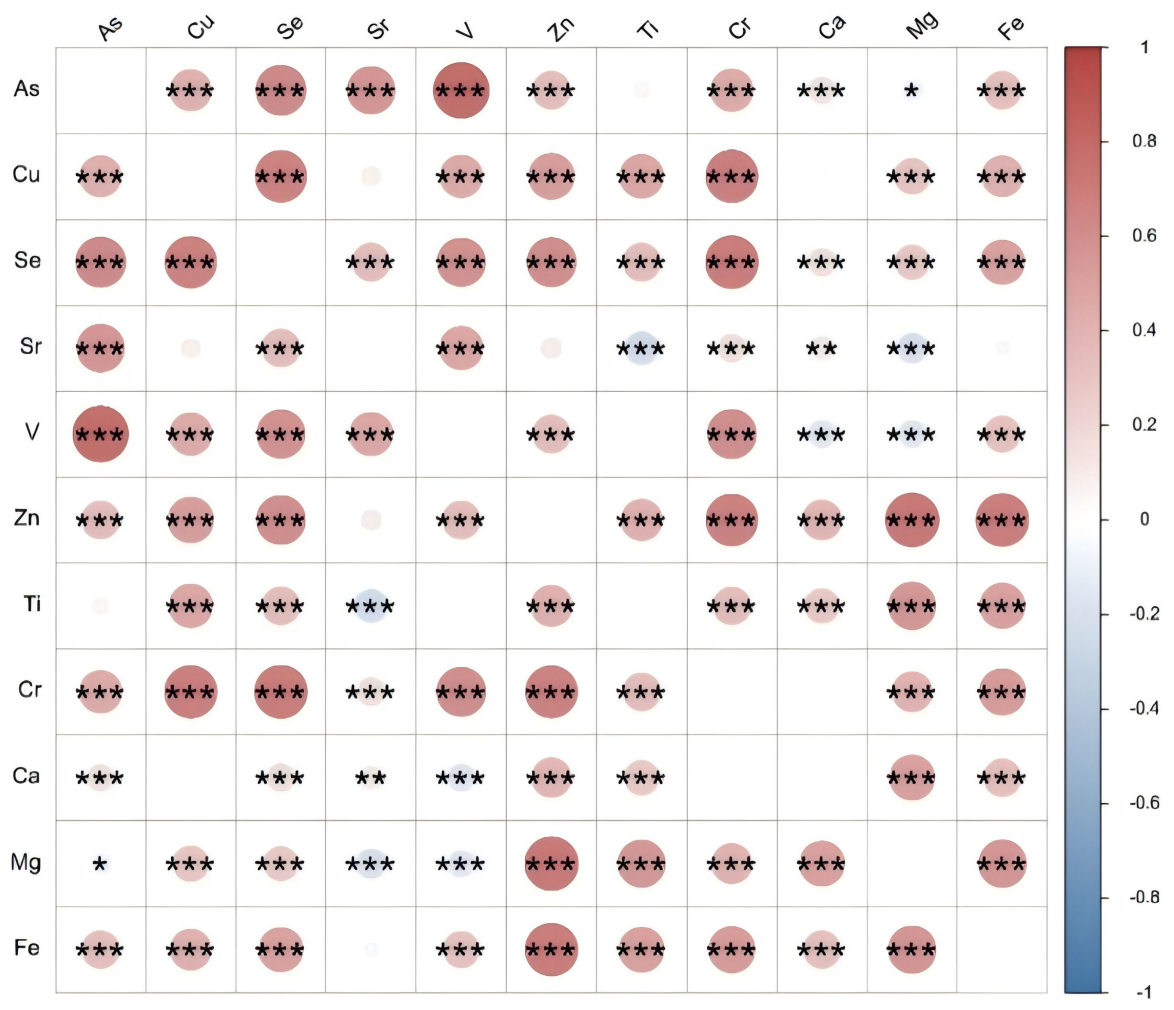
Correlation structure of metal elements in follicular fluid before and after propensity score matching. Pearson correlation matrix of metal concentrations in follicular fluid after propensity score matching. The color intensity and circle size represent the strength and direction of the Pearson correlation coefficient (r), ranging from -1 (negative correlation, blue) to +1 (positive correlation, red). Asterisks indicate statistical significance (*P < 0.05, **P < 0.01, ***P < 0.001).

Spearman correlation analysis in the matched cohort demonstrated significant inter-element relationships (**Figure 1**). Compared with pre-matching results, Sr and Fe, as well as Cr and Ca, showed significant negative correlations (*P* < 0.05). Some previously observed correlations, including those between As and Ca, Se and Sr, and Sr and Cr, were no longer statistically significant after matching.

### 3.7 LASSO and logistic regression analysis

LASSO regression was applied to address multicollinearity and identify key elements associated with DOR. Using 10-fold cross-validation, the optimal penalty parameter (λ) was determined to be 0.0094, and seven elements (As, Cu, Sr, V, Zn, Cr, and Ca) were selected (**Supplementary Figure 2**).

**Figure 2:**
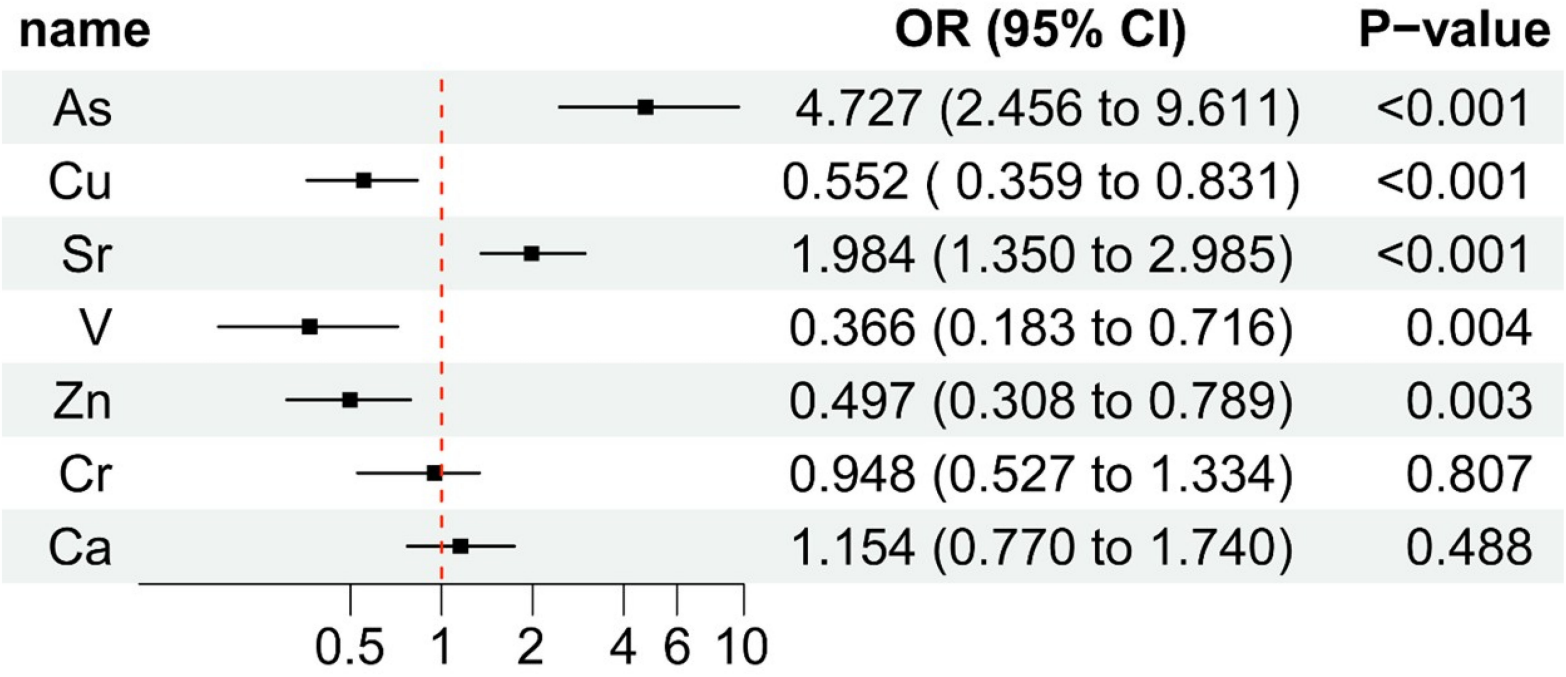
Multivariate logistic regression analysis of metal exposure and diminished ovarian reserve. Forest plot showing the adjusted associations between individual metal elements and the risk of diminished ovarian reserve. Effect estimates are presented as odds ratios (ORs) with 95% confidence intervals (CIs), adjusted for potential confounding variables. Values greater than 1 indicate an increased risk of diminished ovarian reserve, whereas values less than 1 indicate a decreased risk.

Subsequent logistic regression analysis (**Figure 2**) indicated that As (odds ratio [*OR*] = 4.727, 95% confidence interval [*CI*]: 2.456–9.611) and Sr (*OR* = 1.984, 95% *CI*: 1.350–2.985) were associated with higher odds of DOR. In contrast, Cu (*OR* = 0.552, 95% *CI*: 0.359–0.831), V (*OR* = 0.366, 95% *CI*: 0.183–0.716), and Zn (*OR* = 0.497, 95% *CI*: 0.308–0.789) were associated with lower odds of DOR. Meanwhile, the remaining two LASSO-selected elements, Cr (*OR* = 0.948, 95% *CI*: 0.527–1.334) and Ca (*OR* = 1.154, 95% *CI*: 0.770–1.740), were retained in the multivariable model but did not reach statistical significance (*P* > 0.05).

### 3.8 WQS regression analysis

**Figure 3:**
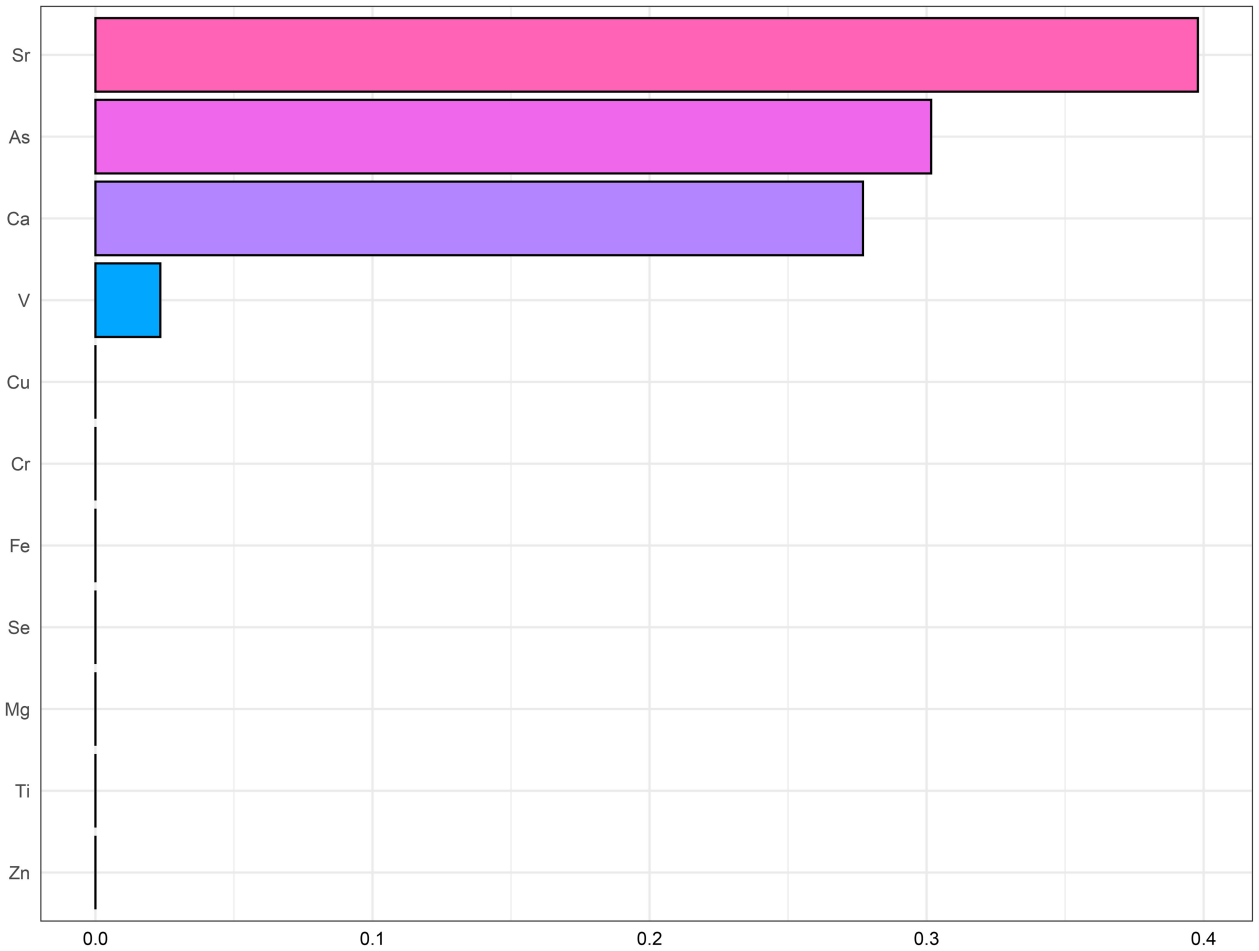
Relative weights of metal elements in the follicular fluid in the WQS model. Relative contribution (weights) of individual metal elements to the overall mixture effect estimated using the WQS regression model. Higher weights indicate a greater contribution of the corresponding metal to the combined effect on diminished ovarian reserve.

WQS regression was used to evaluate the combined effect of metal mixtures. After adjustment for age and BMI, Sr (weight = 0.398), As (weight = 0.302), and Ca (weight = 0.277) contributed most to the mixture index, whereas other elements had negligible contributions (< 0.001) (**Figure 3**).

### 3.9 BKMR analysis

**Figure 4:**
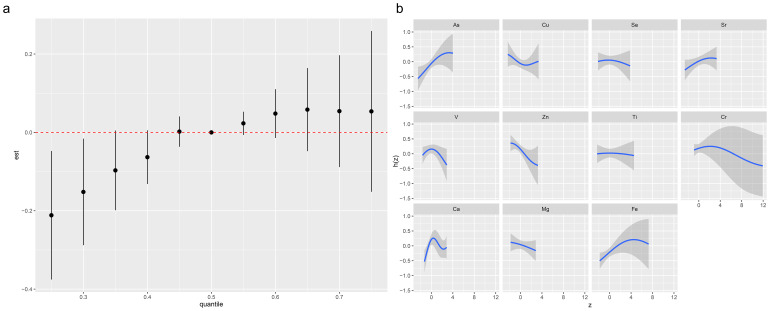
Mixture effects of follicular fluid metal elements on diminished ovarian reserve. **(a)** Quantile-specific effects of the metal mixture. **(b)** Exposure–response curves for individual metal elements.

BKMR was used to assess non-linear and interactive effects of the metal mixture. The overall mixture effect on the likelihood of DOR was positive but relatively weak (**Figure 4**).

Exposure–response analyses indicated that higher levels of As and Sr were associated with increased likelihood of DOR. Cu, V, Ca, and Fe exhibited non-linear relationships, whereas Se, Zn, Cr, and Mg showed inverse associations with DOR.

When other elements were fixed at different percentiles, As, Ca, and Fe remained positively associated with DOR, whereas Zn remained negatively associated (**Supplementary Figure 3**).

### 3.10 Correlation with hormonal indicators

Spearman correlation analysis was conducted to explore associations between selected elements (As, Ca, Fe, and Zn) and hormonal indicators.

As was positively correlated with FSH (*P* < 0.05). No significant association was observed between Ca and hormonal indicators. Fe showed a positive correlation with AMH (*P* < 0.001). Zn was negatively correlated with FSH (*P* < 0.001) and positively correlated with AMH (*P* < 0.001) (**Supplementary Figure 4**).

## 4 Discussion

This case–control study collected baseline characteristics and quantified 11 heavy metals and essential trace elements in FF. We applied a range of analytical approaches to investigate the associations between element exposures in FF and DOR in reproductive-aged women, as well as their relationships with basal hormone levels. After summarizing participant characteristics, we observed a significant age difference between the DOR group and controls, with cases generally older than controls. Given the well-established effects of age and BMI on ovarian function, both factors were adjusted for in subsequent analyses. After adjustment, significant differences were identified in three basal hormones (FSH, T, and AMH) between groups, along with significant differences in multiple measured elements in FF. Correlation analyses further indicated inter-element associations among those that differed significantly, suggesting that these elements may influence the development of DOR through combined effects.

To address limitations of traditional logistic regression—particularly multicollinearity and potential model misspecification, we employed WQS regression and BKMR. The WQS model identified Sr, As, and Ca as the most influential components of the mixture (weights: 0.398, 0.302, and 0.277, respectively), indicating that these elements may play major roles in the development of DOR. The BKMR model was then used to further characterize mixture effects. Results showed that the probability of DOR increased with rising concentrations of heavy metals and essential trace elements in follicular fluid, supporting a joint effect of these exposures, although the overall effect size was modest, consistent with findings by Genard-Walton et al. (13). Compared with traditional logistic regression, BKMR identified As, Ca, and Fe as factors positively associated with DOR, while Zn emerged as a protective factor. Crucially, evaluating the results across different models highlights the vulnerability of traditional linear approaches to both demographic confounding and statistical artifacts in exposome research. A textbook example in our study is V. Prior to matching, V showed an apparent significant difference between groups, which completely disappeared after PSM effectively controlled for age and BMI, confirming the initial finding was driven by demographic confounding. However, when this post-PSM dataset was entered into the post-LASSO multivariate logistic regression, V spuriously re-emerged as a highly significant “protective” factor. This reflects a conditional mathematical artifact (suppressor effect) driven by mutual adjustment among highly correlated metals, rather than a true biological mechanism. Importantly, the advanced BKMR model, which flexibly evaluates the joint mixture surface, correctly did not retain V. These findings underscore the necessity of interpreting traditional logistic ORs with caution and relying on BKMR to mitigate spurious associations and provide robust inference in mixture analyses. Similarly, the stark contrast regarding Sr, which emerged as the top contributor in the WQS model but was not retained in BKMR, warrants deeper methodological interpretation. WQS evaluates the cumulative mixture effect by constructing a unidirectional index. Because Sr is highly correlated with other environmental elements, its dominant WQS weight likely captures the shared variance and collective toxicity of the overall metal mixture. In contrast, BKMR flexibly isolates the independent effect of each element while holding co-exposures constant. The attenuation of Sr’s significance in BKMR elegantly demonstrates that its association with DOR is not an independent biological effect but rather acts as a collinear surrogate for the broader toxic mixture. Consequently, while Sr serves as a valuable macroscopic indicator of the cumulative heavy metal burden, elements such as As, Ca, and isolated free Fe are the true independent drivers disrupting the follicular microenvironment.

Although the precise mechanisms by which heavy metals act as ovarian toxicants remain incompletely understood, accumulating evidence supports their biological plausibility (14). Specifically, recent evidence has demonstrated that heavy metal accumulation in reproductive tissues can induce profound transcriptomic alterations that severely disrupt oxidative stress and cellular homeostasis pathways (15). This strongly reinforces the biological plausibility that the complex metal-DOR associations observed in our study may fundamentally reflect underlying transcriptomic and molecular perturbations within the localized ovarian microenvironment (16). Arsenic exposure is widespread globally through contaminated drinking water and food, yet its impact on female fertility remains underexplored. Some studies have reported no association between arsenic exposure and reproductive outcome (17, 18), whereas others suggest that arsenic induces oxidative stress via reactive oxygen species, leading to follicular damage and impaired ovarian function (19–21). Arsenic exposure has also been associated with decreased serum E2 and T, and increased LH and FSH, potentially mediated by increased DNA methylation in the promoter region of the steroidogenic factor-1 gene. This epigenetic modification may downregulate key steroidogenic proteins (FSHR, StAR, CYP17A1, HSD3B1, CYP19A1) and signaling pathways (PKA–ERK–JNK–cJUN) (22, 23). Of note, heavy metals are also being explored as therapeutic agents, particularly in oncology, though their use remains controversial. For example, approximately one-fifth of female survivors treated with heavy metal-based chemotherapy during childhood exhibited reduced ovarian reserve, suggesting potential gonadotoxicity (24). Conversely, compounds such as As_2_O_3_ and As_4_O_6_ have shown promise as treatments for ovarian cancer, particularly in combination with cisplatin (25). These findings underscore the need to further elucidate the dual roles of arsenic in ovarian physiology and pathology to clarify its toxicity and therapeutic potential. In addition, logistic regression suggested that Sr may be positively associated with DOR, although this association was not supported by BKMR, possibly due to widespread environmental exposure to Sr (e.g., through water) (26). Further research is warranted to clarify the relationship between Sr exposure and ovarian reserve.

Essential trace elements, including Cu, Zn, Ca, and Fe, serve as cofactors in numerous metabolic processes and are critical for normal physiological function (27). Given their involvement in cellular metabolism and antioxidant defense, these essential trace elements play important roles in oocyte development and quality (28). However, evidence regarding their effects on ovarian reserve remains limited. In our study, mixture modeling (BKMR) revealed that elevated Ca levels in the FF were positively associated with the risk of DOR. While systemic Ca is often viewed as protective (29), within the localized ovarian microenvironment, dysregulation of calcium channel signaling and subsequent intracellular calcium overload is a well-established trigger for endoplasmic reticulum stress and mitochondria-dependent apoptosis in granulosa cells (30, 31). The excessive accumulation of Ca in the FF of DOR patients may therefore reflect a severe disruption in local calcium homeostasis, leading to impaired follicular survival. Crucially, with respect to Fe, our findings demonstrated a significant decrease in Fe concentrations in the FF of the DOR group. This contrasts with previous toxicological studies emphasizing systemic iron overload, thereby highlighting the unique metabolic demands of the target microenvironment. Iron is an indispensable cofactor for mitochondrial enzymes involved in oxidative phosphorylation and ATP synthesis during folliculogenesis. A localized iron deficiency in FF can severely impair the cellular energy metabolism of granulosa cells, leading to compromised steroidogenesis and oocyte maturation. Indeed, recent animal models have demonstrated that iron restriction directly impairs follicular development from the secondary to the antral stage, resulting in reduced fertility (32). Consistent with these biological mechanisms, our population-based analysis underscores localized Fe deficiency, rather than overload, as a critical factor associated with DOR. Interestingly, an apparent paradox emerges when comparing the crude elemental profile with the BKMR results. While unadjusted and PSM-matched data indicate a lower Fe concentration in the DOR group, the independent exposure-response function in BKMR identified elevated Fe as a positive associated factor. This discrepancy elegantly highlights the complexity of mixture exposures. In the crude microenvironment, Fe is positively correlated with most other metals, particularly essential trace elements (e.g., Zn, Cu) that are also significantly depleted in DOR patients. Consequently, the overall lower Fe in cases, along with its positive correlation with the ovarian reserve marker AMH, likely reflects a systemic “co-depletion” of essential metabolic cofactors in the pathological state. However, the BKMR model assesses the effect of Fe when other concurrent exposures, including protective antioxidants like Zn, are held constant. Once isolated from this protective trace element network, the independent accumulation of unbuffered free iron is highly gonadotoxic; excess intracellular iron catalyzes the Fenton reaction, generating massive amounts of reactive oxygen species (ROS) and inducing severe oxidative stress in granulosa cells. Therefore, while DOR ovaries suffer from a baseline depletion of essential elements including Fe, the independent over-accumulation of isolated free iron remains an intrinsic risk factor for follicular damage. In contrast, Zn has been shown to promote granulosa cell proliferation, follicular development, and oocyte maturation (33), supporting a beneficial role in ovarian function. Additionally, a positive correlation has been reported between ovarian response and the Cu/Zn ratio in both follicular fluid and plasma (34), suggesting potential synergistic effects between these elements. Consistent with our results, Zn appears to exert a protective effect against DOR, although its underlying mechanisms require further elucidation.

Several limitations should be acknowledged. First, regarding study design and exposure assessment, our case-control approach precludes establishing temporality or causality, and the observed associations might reflect reverse causation driven by altered FF microcirculation in DOR. Furthermore, FF samples were collected at a single time point, and we lacked comprehensive data on potential confounders. Specifically, we were unable to adjust for lifestyle factors (e.g., smoking, alcohol consumption, diet, seafood intake, supplement use), socioeconomic and environmental histories (e.g., occupational exposure, residential area, drinking water source), detailed physiological markers, and specific clinical parameters (e.g., infertility duration, total gonadotropin dose, and the exact number of aspirated follicles). Therefore, our findings primarily reflect localized microenvironmental associations rather than definitive systemic nutritional etiologies (35). Regarding sample representativeness, recruiting women undergoing ICSI for male-factor infertility as controls is an ethically necessary standard, but differences in psychological stress, lifestyle, or unmeasured exposures mean that extrapolating our findings to the general reproductive-age population should be approached with caution. Third, regarding statistical power, while PSM was strictly necessary to eliminate age and BMI confounding, it substantially reduced our sample size from 432 to 242. This reduction inevitably decreased statistical power, meaning borderline significant results should be interpreted cautiously. Finally, regarding sampling methodology and mechanisms, FF samples were pooled rather than analyzed by individual follicle size, potentially masking size-dependent variations. And the lack of matched blood samples precludes evaluating blood–ovarian barrier dynamics (36), and our purely epidemiological observations necessitate future animal and cellular experiments to elucidate the underlying biological mechanisms. Additionally, while visibly blood-contaminated FF samples were strictly excluded, we did not perform biochemical assays (e.g., hemoglobin or erythrocyte counts). Therefore, trace microscopic blood contamination cannot be definitively ruled out, which might subtly influence the quantification of highly blood-enriched elements such as Fe and Ca.

## 5 Conclusion

In summary, this study provides epidemiological evidence supporting an association between metal element mixtures in FF and DOR. The findings underscore the importance of considering combined exposures and highlight the need for further longitudinal and mechanistic studies to clarify the role of environmental metals in ovarian function.

## Data Availability

The raw data supporting the conclusions of this article will be made available by the authors, without undue reservation.
