# Supplementary material for: Association between heavy metals, essential trace elements in follicular fluid and diminished ovarian reserve: a hospital-based case-control study
Source: Front Endocrinol (Lausanne). 2026 Jul 2;17:1889096. doi: 10.3389/fendo.2026.1889096 (PMC13372584; doi:10.3389/fendo.2026.1889096)
Supplement: Supplementary file 1 [file DataSheet1.docx]

**Supplementary table 1. Analytical quality control parameters, detection profiles, descriptive statistics, and final inclusion/exclusion status for all 21 measured elements.**

| **Elements** | **LOD** | **LOQ (μg/L)** | **Spike-recovery percentages (%)** | **Detection Rate (%)** | **Median (IQR)** | **Status** |
| --- | --- | --- | --- | --- | --- | --- |
| As (μg/L) | 0.015 | 0.045 | 101.46 | 100 | 30.89 (27.35, 35.52) | Included |
| Be (μg/L) | 0.003 | 0.009 | 103.73 | 0 | < LOD | Excluded |
| Cd (μg/L) | 0.005 | 0.015 | 97.40 | 0 | < LOD | Excluded |
| Co (μg/L) | 0.024 | 0.072 | 105.88 | 0 | < LOD | Excluded |
| Cu (μg/L) | 0.530 | 1.59 | 104.73 | 100 | 655.25 (522.68, 802.36) | Included |
| Li (μg/L) | 0.028 | 0.084 | 107.33 | 0 | < LOD | Excluded |
| Mo (μg/L) | 0.068 | 0.204 | 102.33 | 0 | < LOD | Excluded |
| Mn (μg/L) | 0.938 | 2.814 | 107.37 | 53.4 | 1.29 (-2.42, 4.52) | Excluded |
| Ni (μg/L) | 5.036 | 15.108 | 103.33 | 0 | < LOD | Excluded |
| Pb (μg/L) | 0.176 | 0.528 | 99.87 | 35.4 | -0.49 (-8.8, 0.63) | Excluded |
| Sb (μg/L) | 0.148 | 0.444 | 98.16 | 52.3 | 0.17 (-0.01, 0.99) | Excluded |
| Se (μg/L) | 0.201 | 0.603 | 98.17 | 100 | 71.48 (56.47, 91.85) | Included |
| Sr (μg/L) | 0.449 | 1.347 | 103.76 | 100 | 32.6 (24.06, 41.1) | Included |
| Tl (μg/L) | 0.003 | 0.009 | 101.26 | 10.3 | -0.04 (-0.01, 0.08) | Excluded |
| V (μg/L) | 0.060 | 0.18 | 104.60 | 100 | 29.51 (23.79, 37.41) | Included |
| Zn (μg/L) | 4.949 | 14.847 | 80.79 | 100 | 319.04 (148.79, 442.71) | Included |
| Ti (μg/L) | 0.995 | 2.985 | 103.52 | 100 | 163.37 (136.56, 191.48) | Included |
| Cr (μg/L) | 0.7088 | 2.1264 | 104.50 | 100 | 91.74(73.35, 112.67) | Included |
| Ca (mg/L) | 0.937 | 2.811 | 114.10 | 100 | 55.82 (45.77, 68.62) | Included |
| Mg (mg/L) | 0.082 | 0.246 | 119.14 | 100 | 36.80 (28.40, 46.06) | Included |
| Fe (μg/L) | 68.278 | 204.834 | 118.43 | 100 | 1441.08 (984.53, 1840.48) | Included |

**Supplementary table 2. Baseline Characteristics of Participants After Propensity Score Matching**

| **Variables** | **Control (N=121)** | **DOR (N=121)** | **P-value** |
| --- | --- | --- | --- |
| BMI (kg/m^2) | 21.55 (19.94, 23.38) | 21.45 (19.64, 23.31) | 0.469 |
| Age (years) | 33.00 (30.00, 36.00) | 33.00 (30.00, 36.00) | 0.998 |
| No. of retrieved oocytes | 9.00 (7.00, 13.00) | 4.00 (3.00, 6.00) | <0.001 |
| FSH (mIU/mL) | 6.29 (5.30, 7.60) | 7.33 (6.14, 9.67) | <0.001 |
| LH (mIU/mL) | 3.30 (2.60, 4.38) | 3.10 (2.25, 4.40) | 0.279 |
| PRL (ng/mL) | 14.00 (10.60, 20.60) | 14.40 (10.28, 19.98) | 0.932 |
| E2 (pg/mL) | 36.00 (29.00, 48.00) | 36.00 (25.00, 46.00) | 0.587 |
| T (ng/dL) | 0.28 (0.23, 0.34) | 0.25 (0.20, 0.33) | 0.005 |
| AMH (ng/mL) | 2.73 (2.08, 4.58) | 0.80 (0.58, 1.27) | <0.001 |

Data are presented as Median (25th Percentile, 75th Percentile).

Abbreviations: FSH, Follicle-Stimulating Hormone; LH, Luteinizing Hormone; PRL, Prolactin; E2, Estradiol; T, Testosterone; AMH, Anti-Müllerian Hormone

**Supplementary table 3. Exposure Levels of Heavy Metals and Essential Trace Elements in Follicular Fluid After Propensity Score Matching**

| **Element** | **Control (N=121)** | **DOR (N=121)** | ***P*** |
| --- | --- | --- | --- |
| As (μg/L) | 30.77 (27.92, 37.10) | 33.79 (30.81, 36.78) | 0.001 |
| Cu (μg/L) | 752.57 (609.22, 879.45) | 609.84 (493.64, 782.79) | <0.001 |
| Se (μg/L) | 76.89 (63.22, 98.09) | 77.79 (63.95, 92.11) | 0.336 |
| Sr (μg/L) | 30.68 (23.78, 38.39) | 38.46 (32.00, 46.48) | <0.001 |
| V (μg/L) | 30.92 (23.83, 48.08) | 30.70 (27.98, 38.15) | 0.843 |
| Zn (μg/L) | 425.79 (314.99, 499.63) | 267.43 (149.28, 401.25) | <0.001 |
| Ti (μg/L) | 168.61 (144.12, 193.59) | 158.90 (122.79, 192.43) | 0.035 |
| Cr (μg/L) | 104.83 (87.09, 132.82) | 88.26 (70.82, 110.28) | <0.001 |
| Ca (mg/L) | 50.94 (44.40, 66.78) | 57.94 (53.98, 67.21) | <0.001 |
| Mg (mg/L) | 40.99 (34.01, 46.76) | 30.26 (22.88, 43.87) | <0.001 |
| Fe (μg/L) | 1574.11 (1229.91, 1976.31) | 1476.73 (1058.94, 1784.20) | 0.038 |

Data are presented as Median (25th Percentile, 75th Percentile).

Abbreviations: As, Arsenic; Cu, Copper; Se, Selenium; Sr, Strontium; V, Vanadium; Zn, Zinc; Ti, Titanium; Cr, Chromium; Ca, Calcium; Mg, Magnesium; Fe, Iron.


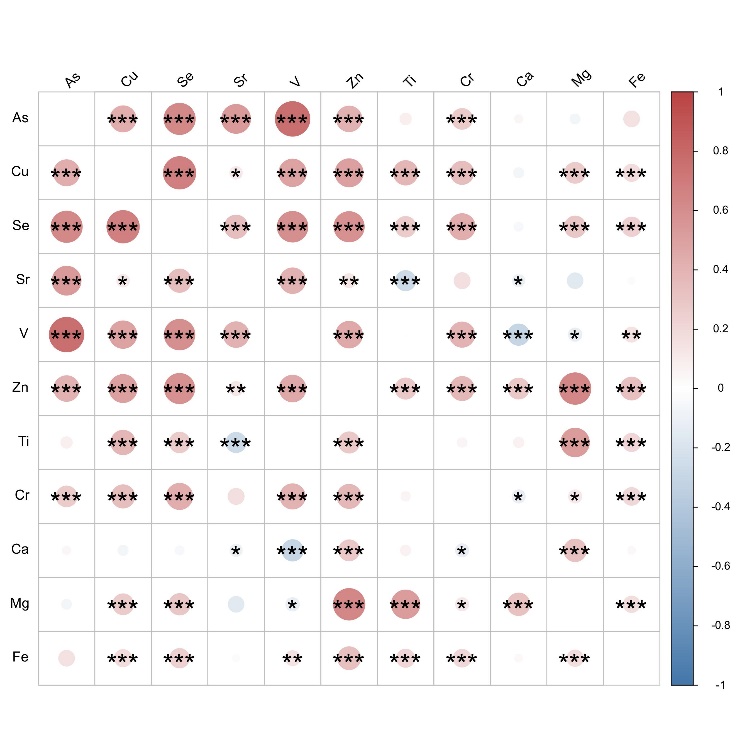


**Supplementary** **figure 1.** Correlation structure of metal elements in follicular fluid before and after propensity score matching. Pearson correlation matrix of metal concentrations in follicular fluid before propensity score matching. The color intensity and circle size represent the strength and direction of the Pearson correlation coefficient (r), ranging from -1 (negative correlation, blue) to +1 (positive correlation, red). Asterisks indicate statistical significance (*P < 0.05, **P < 0.01, ***P < 0.001).


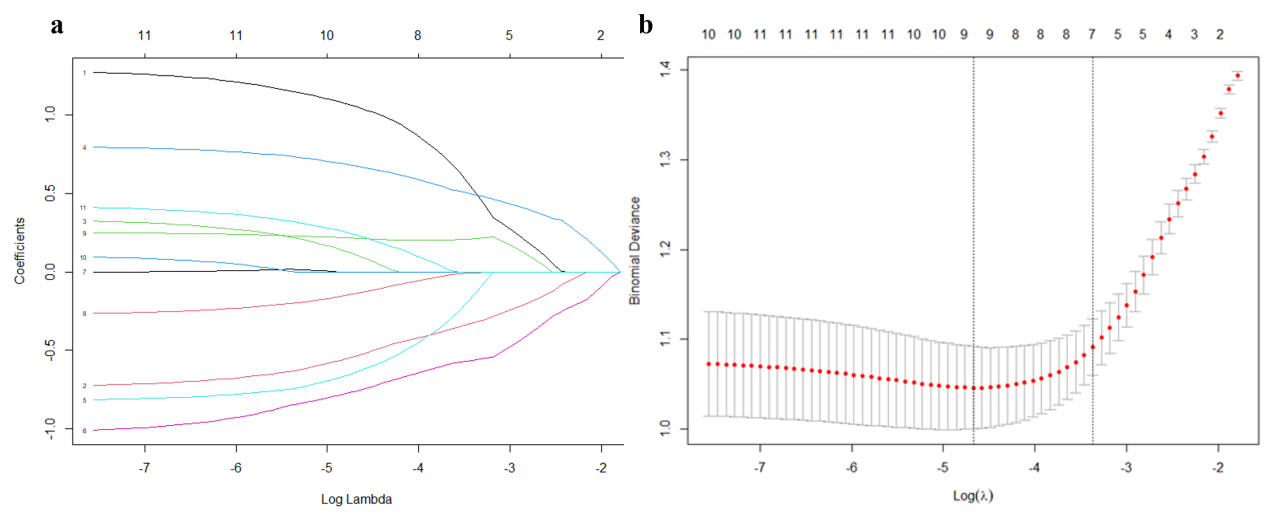


**Supplementary** **figure 2.** Least absolute shrinkage and selection operator (lasso) model for variable selection. (a) LASSO regression coefficient path plot. (b) Cross-validation deviance plot for selection of the optimal penalty parameter (λ).


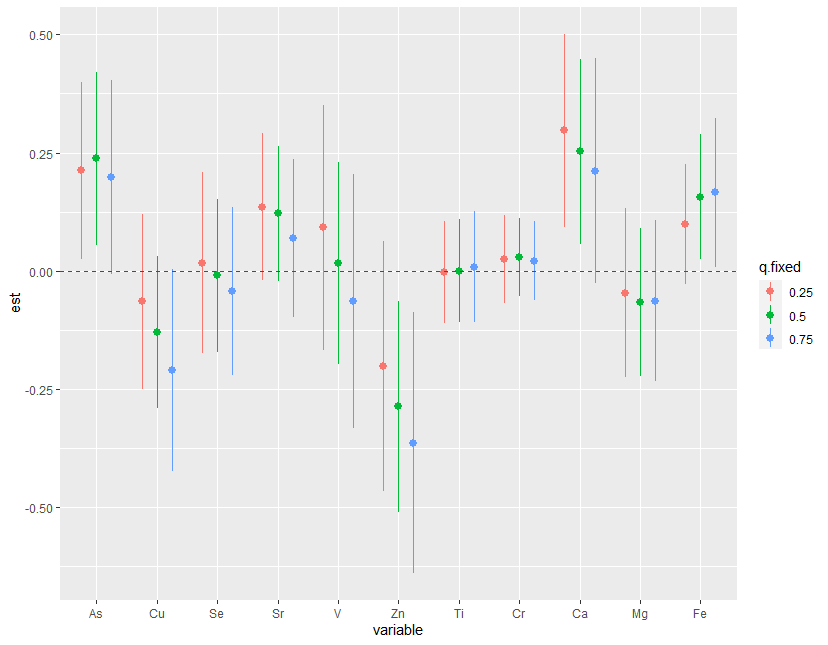
**Supplementary** **figure 3.** Conditional effect estimates of individual metals at different background exposure levels. Panel

**
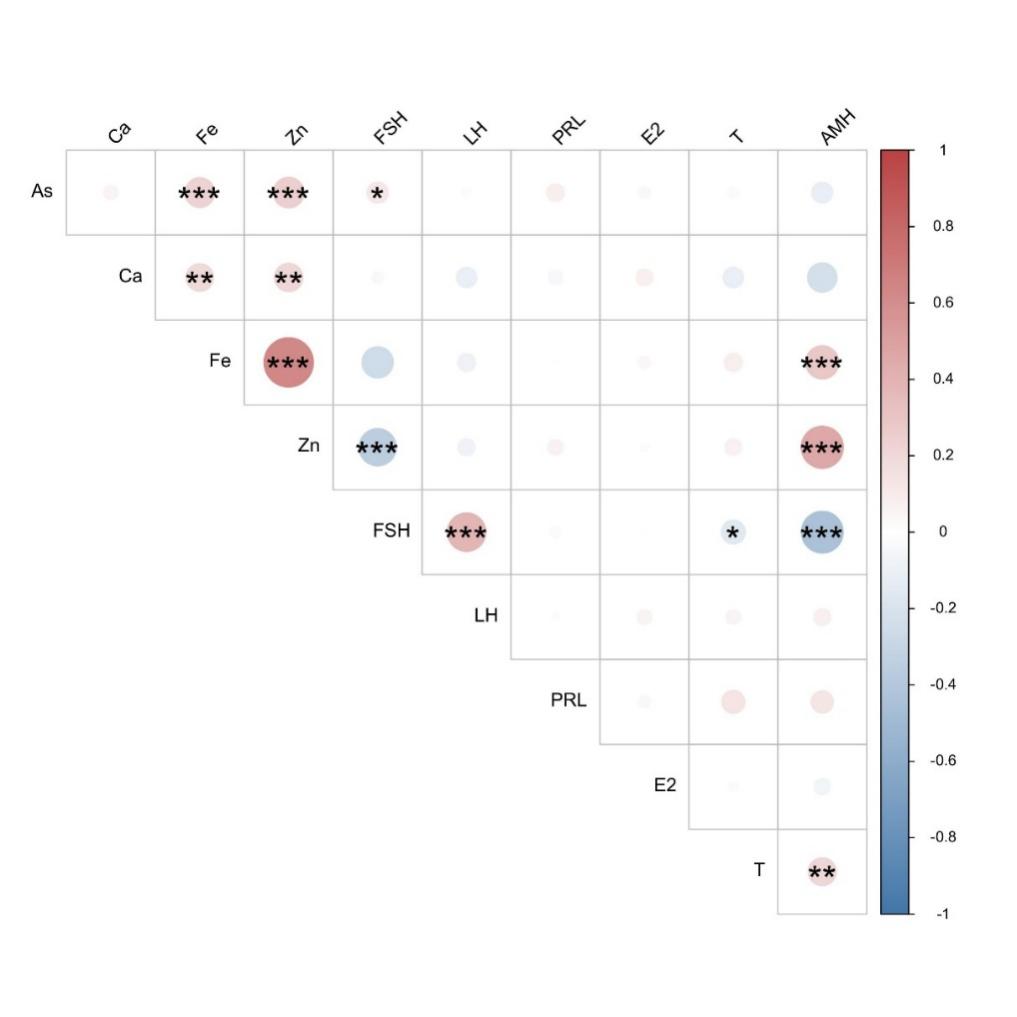
**

**Supplementary** **figure 4.** Correlation Between Metal Elements and Basal Hormones in Follicular Fluid Pearson correlation matrix illustrating the relationships between selected metal elements (arsenic, calcium, iron, and zinc) and basal hormone levels in follicular fluid. The color intensity and circle size represent the magnitude and direction of the correlation coefficient (r), ranging from −1 (blue) to +1 (red). Asterisks indicate statistical significance (*P < 0.05, **P < 0.01, ***P < 0.001).
